# Complete mitochondrial genome of *Ostrinia kasmirica* (Lepidoptera: Crambidae)

**DOI:** 10.1080/23802359.2021.1950058

**Published:** 2021-07-09

**Authors:** Qiuyu Luo, Nan Zhou, Zhaofu Yang

**Affiliations:** a Key Laboratory of Plant Protection Resources and Pest Management, Ministry of Education, Northwest A&F University, Yangling, PR China; b Entomological Museum, College of Plant Protection, Northwest A&F University, Yangling, PR China

**Keywords:** Mitochondrial genome, Crambidae, *Ostrinia*, phylogenetic analysis

## Abstract

The complete mitochondrial genome of *Ostrinia kasmirica* (Moore, 1888) was sequenced in this study. The circular mitogenome is 15,214 bp in length, containing 37 typical encoded genes and a non-coding control region. The gene organization and nucleotide composition are similar to those of most other sequenced *Ostrinia* species. All protein-coding genes (PCGs) initiate with ATN and terminate with TAN, except *cox1* starts with CGA and *cox1*, *cox2*, *nad5* terminate with an incomplete codon T. The control region of 308 bp contains three conserved features including the motif ‘TTAGA’ preceded a poly-T stretch, a microsatellite-like (TA)_n_ element, and a poly-A stretch upstream of *trnM*. Phylogenetic analysis based on mitogenome sequences revealed that the *O. kasmirica* (the second species group) was more closely related to the third species group of the genus and the first species group was not at the basal position of this genus as that Mutuura and Munroe indicated.

The genus *Ostrinia* Hübner, 1825 (Crambidae: Pyraustinae) includes 23 species, with a few well-known destructive pests that caused huge economic losses (Mutuura and Munroe 1970). Mutuura and Munroe (1970) recognized three species groups of the genus based on the morphology of male genitalia. The first species group (Group I) only contains a single species *Ostrinia* *penitalis* (Grote, 1876) with unarmed sacculus and one-lobed uncus which is regarded as the ‘primitive’ species of this genus. The second species group (Group II) includes nine species with simple or weakly bifid uncus in the male genitalia. The third species group (Group III) comprises 10 species such as *Ostrinia furnacalis* (Guenée, 1854) and *Ostrinia nubilalis* (Hübner, 1796) . The remaining species including *Ostrinia maysalis* Leraut, 2012, *Ostrinia ovalipennis* Ohno, 2003, and *Ostrinia avarialis* Amsel, 1970 not classified into the above three species groups.

The *Ostrinia kasmirica* (Moore, 1888) is commonly found in the northeast India, southeast Russia, and Siberia (Mutuura and Munroe 1970). It was first recorded by Li and Tang in 1981 in China (Li and Tang 1985). The larva of this species mainly feeds on the thistle *Cnicus wallichi*. Mutuura and Munroe (1970) purported that the *O. kasmirica*, having bell-shaped and weakly bifid uncus, is most likely to be closely related with *Ostrinia latipennis* (Warren, 1892) and *Ostrinia* *palustralis* (Hübner,1796) based on the male genitalia within the second species group. However, the *O. kasmirica* is similar to *O. nubilalis* and *Ostrinia scapulalis* (Walker, 1859) in appearance (Bidzilya and Budashkin 2017), implying that *O. kasmirica* is closely related to the members of the third species group. Due to the lack of the molecular data of *O. kasmirica* its phylogenetic position is still unclear. To address this question, we sequenced the complete mitogenome sequence of *O. kasmirica* and inferred the phylogenetic relationships of this genus based on this mitogenome and other six published mitogenome sequences of the genus *Ostrinia* .

The adult specimens of *O. kamirica* were collected by light trap in July 2018 from Jalai Nur District (49°31'N, 117°43'E), Hulunbuir Prefecture, Inner Mongolia Autonomous Region, China. Specimens were preserved in a freezer in pure ethanol at −20 °C. The voucher sample was deposited at the Entomological Museum (Voucher number ZLNE3), Northwest A&F University, Yangling, Shaanxi, China. Genomic DNA was extracted from thoracic muscle. Illumina HiSeq platform (Illumina, San Diego, CA) was used for sequencing and generated 2 × 150 bp paired-end reads. Mitochondrial gene annotation was performed by MitoZ version 2.4 (Meng et al. 2019) under all module which includes four steps: raw data pretreatment, *de novo* assembly with SOAPdenvo-Trans, mitogenome sequence identification, and mitogenome annotation . Before data analyses, the annotated mitogenome was rechecked with previously published mitogenomes of *Ostrinia* species.

The complete mitogenome of *O. kasmirica* (GenBank accession number. MT978075) is circular molecular structure of 15,214 bp in length, containing 37 typical genes (13 PCGs, 22 transfer RNAs and 2 ribosomal RNAs) and a non-coding control region. The gene arrangement and content are identical to most of the mitogenomes in Lepidoptera. Nucleotide compositions of 41.8% A, 39.2% T, 11.3% C, and 7.7% G indicated that the *O. kasmirica* mitogenome is biased toward higher A + T content. All PCGs initiate with standard start codon ATN except *cox1* with the special start codon CGA. Most PCGs terminate with TAN, whereas *cox1*, *cox2*, and *nad5* stop with an incomplete codon T. All tRNAs exhibit cloverleaf secondary structure except *trnS1(AGN)* which was missing the dihydrouridine (DHU) arm. The length of *12S rRNA* and *16S rRNA* are 777 and 1,339 bp, respectively. The control region of the *O. kasmirica* is 308 bp in length with several conserved blocks, including a ‘TTAGA’ motif proceeding a 19-bp poly-T stretch, a microsatellite-like (TA)_n_ element, and an ‘A-rich’ upstream of *trnM,* which are commonly found in other published mitogenomes of the genus *Ostrinia* (Zhou et al. 2020).

Phylogeny of the genus *Ostrinia* was inferred based on the concatenated nucleotide sequences of 13 PCGs with all codon positions using both maximum likelihood (ML) and Bayesian inference (BI) methods. Best substitution model for ML and BI was selected based on PartitionFinder version 2.1.1, respectively (Lanfear et al. 2016). There are seven partition schemes for the ML analysis (Subset 1: GTR + I, *atp6*_pos1, *cox1*_pos1, *cox2*_pos1, *cox3*_pos1, *cytb*_pos1; Subset 2: HKY + I, *atp6*_pos2, *cox1*_pos2, *cox2*_pos2, *cox3*_pos2, *cytb*_pos2; Subset 3: GTR + I+G, *atp6*_pos3, *atp8*_pos3, *cox1*_pos3, *cox2*_pos3, *cox3*_pos3, *cytb*_pos3, *nad2*_pos3, *nad3*_pos3, *nad6*_pos3; Subset 4: GTR + I, *atp8*_pos1, *atp8*_pos2, *nad2*_pos1, *nad3*_pos1, *nad6*_pos1; Subset 5: HKY + I, *nad1*_pos1, *nad4*_pos1, *nad4L*_pos1 *nad5*_pos1; Subset 6: HKY + I, *nad1*_pos2, *nad2*_pos2, *nad3*_pos2, *nad4*_pos2, *nad4L*_pos2, *nad5*_pos2, *nad6*_pos2; Subset 7: GTR + G, *nad1*_pos3, *nad4*_pos3, *nad4L*_pos3, *nad5*_pos3) and seven partition schemes for the BI analysis (Subset 1: TRN + I, *atp6*_pos1, *cox1*_pos1, *cox2*_pos1, *cox3*_pos1, *cytb*_pos1; Subset 2: HKY + I, *atp6*_pos2, *cox2*_pos2, *cytb*_pos2, *nad1*_pos2, *nad2*_pos2, *nad3*_pos2, *nad4*_pos2, *nad4L*_pos2, *nad5*_pos2, *nad6*_pos2; Subset 3: TIM + I+G, *atp6*_pos3, *atp8*_pos3, *cox1*_pos3, *cox2*_pos3, *cox3*_pos3, *cytb*_pos3, *nad2*_pos3, *nad3*_pos3, *nad6*_pos3; Subset 4: TIM + I, *atp8*_pos1, *nad2*_pos1, *nad3*_pos1, *nad6*_pos1; Subset 5: TRN + I+G, *atp8*_pos2, *nad1*_pos1, *nad4*_pos1, *nad4L*_pos1, *nad5*_pos1; Subset 6: F81 + I, *cox1*_pos2, *cox3*_pos2; Subset 7: GTR + G, *nad1*_pos3, *nad4*_pos3, *nad4L*_pos3, *nad5*_pos3). Six species within the genus *Ostrinia* were selected as ingroups (Coates and Abel 2016; Hwang et al. 2019; Zhou et al. 2020). *Loxostege sticticalis* (Linnaeus, 1761) (Crambidae: Pyraustinae) and *Cnaphalocrocis medinalis* (Guenée, 1854) (Crambidae: Spilomelinae) were selected as outgroups (Wan et al. 2013; Ma et al. 2016). The ML analyses were performed using IQ-TREE version 1.6.8 (Nguyen et al. 2015) and BI analyses were conducted with MrBayes version 3.2.6 (Ronquist et al. 2012 ). The phylogenetic topologies of the ML and BI analyses agreed with each other (Figure 1). The *O. kasmirica* (Group II) and four species including *O. zealis*, *O. furnacalis*, *O. nubilalis*, and *O. scapulalis* of the third species group were clustered into a clade with high nodal support values, indicating *O. kasmirica* is likely to be more closely related to the trifid-uncus species group other than *O. palustralis* (Group II). Our results confirmed that the *O. penitalis* (Group I) is not the primitive species among the genus *Ostirnia,* which is consistent with the previous study (Zhou et al. 2020).

**Figure 1.**
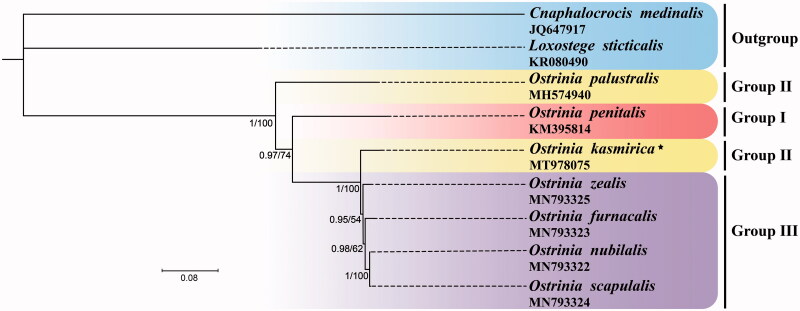
Phylogenetic trees using ML analyses based on 13 PCGs of nine mitogenomes. BI analyses show the same topology (not shown). The numbers under the branches are Bayesian posterior probabilities (PP) and bootstrap support values (BS). Alphanumeric terms indicate the GenBank accession numbers.

## Data Availability

The data that support the findings of this study are openly available in GenBank at https://www.ncbi.nlm.nih.gov, reference number MT978075. The associated BioProject accession number, SRA data, and BioSample accession number are PRJNA716609, SRR14044899, and SAMN18439942 respectively.
